# Foreign Items

**Published:** 1869-03-01

**Authors:** 


### FOREIGN ITEMS.

Dr. Paul Niemeyer, of Magdebourg, proposes a new stethescope to which he applies the name acouoxylon. Referring to the manner in which the stethescope was discovered by Laennec, and to the fact that the scratch of a pin made upon one extremity of a beam may be distinguished by the ear applied to the other end of the same, he asserts that the distinguished inventor fell into a radical error in making use of a hollow cylinder of paper, and subsequently of wood, constituting a medium compounded of paper (or wood) and air, and hence possessing different capacities for conducting sound. Although, he continues, Laennec himself was astonished and satisfied at hearing the pulsations of the heart in a much more clear and distinct manner, his successors, on the contrary, have determined that with the ear applied immediately, the same end was accomplished, and that the perception of sounds by the stethescope was even less clear. Moreover, it is certain that Laennec with his cylinder heard many phenomena which have not been confirmed subsequently, and which probably were secondary sounds produced in the interior of the stethescope, which, from the time of Laennec has been of considerable length.

If now we bear in mind the physical law exactly determined by Chladni and Savart, that wood is a much more efficient conductor of sound than air, that in the relation of this capacity pine wood is eighteen times superior to air; in musical instruments, pine wood in *masses* is used whenever the conducting of sound only is desired. We ask, therefore, why there should be used in medicine a hollow cylinder which weakens, and even disturbs the perception of the phenomena which it is desirable to recognize as clearly as possible. The Doctor advises the construction of a stethescope by turning a solid cylinder from a single piece of very dry pine wood, which he terms acouoxylon, and which he asserts to be the only stethescope constructed in accordance with physical laws, and consequently superior in every respect to the stethescope of Laennec. *Probatum est!*

